# Assessment of *In Vivo* and *In Vitro* Genotoxicity of Glibenclamide in Eukaryotic Cells

**DOI:** 10.1371/journal.pone.0120675

**Published:** 2015-03-24

**Authors:** Juliane Rocha de Sant’Anna, Claudinéia Conationi da Silva Franco, Paulo Cezar de Freitas Mathias, Marialba Avezum Alves de Castro-Prado

**Affiliations:** 1 Departamento de Biotecnologia, Genética e Biologia Celular, Laboratório de Genética de Microorganismos e Mutagênese, Universidade Estadual de Maringá, Maringá, Paraná, Brazil; 2 Departamento de Biotecnologia, Genética e Biologia Celular, Laboratório de Biologia Celular e Secreção, Universidade Estadual de Maringá, Maringá, Paraná, Brazil; Wayne State University School of Medicine, UNITED STATES

## Abstract

Glibenclamide is an oral hypoglycemic drug commonly prescribed for the treatment of type 2 diabetes mellitus, whose anti-tumor activity has been recently described in several human cancer cells. The mutagenic potential of such an antidiabetic drug and its recombinogenic activity in eukaryotic cells were evaluated, the latter for the first time. The mutagenic potential of glibenclamide in therapeutically plasma (0.6 μM) and higher concentrations (10 μM, 100 μM, 240 μM and 480 μM) was assessed by the *in vitro* mammalian cell micronucleus test in human lymphocytes. Since the loss of heterozygosity arising from allelic recombination is an important biologically significant consequence of oxidative damage, the glibenclamide recombinogenic activity at 1 μM, 10 μM and 100 μM concentrations was evaluated by the *in vivo* homozygotization assay. Glibenclamide failed to alter the frequency of micronuclei between 0.6 μM and 480 μM concentrations and the cytokinesis block proliferation index between 0.6 μM and 240 μM concentrations. On the other hand, glibenclamide changed the cell-proliferation kinetics when used at 480 μM. In the homozygotization assay, the homozygotization indices for the analyzed markers were lower than 2.0 and demonstrated the lack of recombinogenic activity of glibenclamide. Data in the current study demonstrate that glibenclamide, in current experimental conditions, is devoid of significant genotoxic effects. This fact encourages further investigations on the use of this antidiabetic agent as a chemotherapeutic drug.

## Introduction

Sulphonylurea is a class of oral antidiabetic agents with long clinical use in patients with type 2 diabetes. These agents are insulin secretagogues which act directly on the pancreatic β cell ATP-sensitive potassium channels (K_ATP_ channels) and augment its closure by glucose [1]. The K_ATP_ channels are protein channels that regulate the transport of potassium ions through cell membranes. They are hetero-octameric complexes regulated by the intracellular levels of ATP/ADP ratio and consist of eight subunits arranged in two rings: an inner ring of four inwardly rectifying K^+^ channel (Kir6.X) subunits which form the pore through which potassium ions pass, and an outer ring that comprises four subunits of the regulatory sulphonylurea receptor (SUR) [1–3].

Several therapeutic agents may affect the K_ATP_ channels’ activity. Whereas nicorandil, a drug used for angina pectoris, activates the K_ATP_ channels, the sulphonylureas, *e*.*g*., glibenclamide, used to control type 2 diabetes, inhibit the K_ATP_ channels by their interaction with the SUR subunit. This inhibition leads to β cell membrane depolarization which results in the opening of voltage-gated Ca^2+^ channels and in the induction of Ca^2+^ transport from the extracellular compartment into the cytoplasm of the β cell. A rise in the cytosolic calcium ion concentration linearly increases the exocytosis of the insulin-containing granules into the plasma compartment [1–3].

Previous studies have shown that potassium channels regulate the growth and proliferation of many types of cell, so that K_ATP_ channels blockers, such as glibenclamide, lead to cell proliferation inhibition, whereas openers of K_ATP_ channels produce a hyperpolarization of membrane potential and activate the cell progression through the mitosis’s G1 phase. Since glibenclamide has been shown to inhibit cellular proliferation in several cancer lines, this antidiabetic drug represents a potentially useful compound for cancer treatment [4–6].

Epidemiologic reports, designed to assess the association of malignancies with the use of glibenclamide, gliclazide and other secretagogues for type 2 diabetes treatment, have shown conflicting results. The positive association between mortality for malignancies and secretagogues users was higher for glibenclamide than for gliclazide or tolbutamide [7, 8]. Although glibenclamide has been previously associated with increased cancer risk, a potential protective effect was assigned to gliclazide [9]. On the other hand, the use of glibenclamide and gliclazide, but not glipizide, was associated with reduced cancer risk in a dose-dependent manner in a clinic-based study [10]. Despite these conflicting outcomes, a potential new role for glibenclamide as a chemotherapeutic agent in cancer treatment has been proposed [9, 11, 12].

The anti-tumor action of glibenclamide alone or in combination with tamoxifen was observed on experimental mammary tumors induced by N-nitroso-N-methylurea (NMU) in nondiabetic rats [13]. Furthermore, this K_ATP_ channel blocker was able to inhibit cell invasion and migration of ovarian clear cell carcinoma ES-2 cells by the inhibition of the secretory mechanism of the platelet-derived growth factor AA, involved in multiple tumor-associated processes [11].

Many agents used in cancer chemotherapy have been proposed as reponsible for the second malignances diagnosed in cancer patients after chemotherapeutic regimens. In fact, second malignances are acknowledged as severe long-term consequences of cytotoxic therapies for a primary disorder [14–16]. Since DNA mutation and somatic recombination play important roles in the tumorigenic process [17, 18], the current research assesses the mutagenic and recombinogenic potentials of glibenclamide since it shows anticancer and anti-proliferative properties and is widely used to treat type 2 diabetes mellitus [1–6].

Since glibenclamide has been shown to increase the production of reactive oxygen species (ROS) in β cells [19], and that ROS are known to cause a variety of chemical modifications to nucleic acids, which result in DNA damage [20], current study investigates the mutagenic potential of the sulphonylurea glibenclamide in human lymphocytes by the *in vitro* mammalian cell micronucleus (MNvit) test. The MNvit is a well-established screening method which detects the clastogenic and aneugenic effects of chemical compounds in mammalian cells [21, 22]. Further, assuming that the loss of heterozygosity (the loss of the functional allele at a heterozygous locus) arising from allelic recombination is one important biologically significant consequence of the oxidative damage [23], the glibenclamide recombinogenic potential was also evaluated by the *in vivo* homozygotization assay, a sensitive, low-cost and rapid eukaryotic test which detects mitotic recombination events in diploid cells of *Aspergillus nidulans* [24].

## Materials and Methods

### 1. Chemicals and reagents

Glibenclamide (Sigma-Aldrich, CAS N° 10238–21–8) was used as the test substance for *in vitro* and *in vivo* tests. Glibenclamide was dissolved in 1% dimethyl sulfoxide (DMSO CAS n° 67–68–5) supplied by Sigma-Aldrich (St.Louis, Mo, USA), which was also tested as solvent control. Mitomycin-C (CAS n° 50–07–7, St. Louis, MO), used as the positive control for the MNvit test, cytosine arabinoside (CAS n° 205–705–9, St. Louis, MO), used in the *in vivo* homozygotization assay, and cytochalasin B (CAS n° 14930–96–2, St. Louis, MO) were also purchased from Sigma. RPMI 1640 cell culture media supplemented with L-glutamine (11875–093), fetal bovine serum (12657–029) and phytohemagglutinin (10576–015) were purchased from Gibco Life Technologies. Giemsa and all other chemicals were purchased from Merck (Darmstadt, Germany). All test solutions were freshly prepared before each experiment. All chemicals, solvents and culture media used in this study were of the highest purity.

### 2. Lymphocyte isolation

Following OECD guideline 487 [25] and Vlastos et al. [26], current study was carried out using human peripheral blood samples from two healthy, non-smoking volunteer donors aged 20 to 25 years. All donors had no previously known exposure to high concentrations of genotoxicants. All volunteers gave their informed consent to participate in the study and signed the consent forms. Current study was approved by the Ethics Committee of the Universidade Estadual de Maringá, Maringá PR Brazil. Freshly collected, heparinised peripheral blood was used. After centrifugation at 1100 rpm for 5 min, the lymphocyte layer was collected and added to 82% RPMI 1640 medium supplemented with 15% fetal bovine serum, 1% L-glutamine 200 mM and 2% phytohemagglutinin.

### 3. Selection of glibenclamide testing concentrations

For the selection of glibenclamide concentrations, the effects of the wide range of concentrations were evaluated using the mitotic index (MI) as a cytotoxicity marker. While the highest glibenclamide concentration selected produced 55 ± 5% cytotoxicity [25], the lowest concentration employed was the glibenclamide plasma concentration (0.6 μM) [27]. MI was calculated as follows [28]:

$$\text{MI=}\frac{\text{number of dividing cells}}{\text{total numbr of the cells}}\times 100$$

### 4. MNvit test [25]

The glibenclamide plasma concentration (0.6 μM) and four higher concentrations (10 μM, 100 μM, 240 μM and 480 μM) were used in the MNvit test. The lymphocyte cultures were incubated at 37°C in a humidified atmosphere with 5.0% CO_2_, for 72 h. The cells were treated with glibenclamide and mitomycin-C (0.3 μM, positive control) at 24 h after initiating the culture. Cytochalasin B (final concentration of 12.5 μM) was added after 44 h of incubation in order to block cytokinesis and obtain binucleated cells (BC). After an additional 28 h incubation at 37°C, the cells were harvested by mild centrifugation and the pellet was resuspended in a cold hypotonic solution of 75 mM KCl. The cells were fixed in a cold fixative solution (methanol: glacial acetic acid, 3:1 v/v) and, after mild centrifugation, they were fixed thrice with methanol: glacial acetic acid (3:1 v/v). In the first fixative solution, 1% formaldehyde was added to preserve the cytoplasm. Slides were prepared by dropping and air drying. The slides were stained with 5% Giemsa solution (diluted with Sorensen buffer, pH 6.8) for 7 min [25, 29]. To determine the number of micronuclei and other nuclear anomalies, the lymphocytes cultures were performed in duplicates per each donor, with 1000 BC with preserved cytoplasm scored per culture and for each treatment (4000 BC were scored per concentration, 2000 BC for each donor). The cytokinesis block proliferation index (CBPI) was determined as follows:

$$\text{CBPI= }\frac{\text{N1+2 N2+3 }\left(\text{N3+N4}\right)}{\text{500}}$$

where N1 to N4 are the cells with one to four nuclei in 500 cells counted for each experiment [30]. CBPI indicates the average number of cell cycles per cell during the period of exposure to cytochalasin B and may be used to calculate cell proliferation [25]. The experiments were done in duplicates for each donor. The amount of cytostasis (or inhibition of cell growth, [25]) induced by each treatment was determined as follows:

$$\text{\% cytostasis=100-100 }\left[\frac{{\text{CBPI}}_{\text{T}}\text{-1}}{{\text{CBPI}}_{\text{C}}\text{-1}}\right]$$

where CBPI_T_ is determined in each treatment and CBPI_C_ is determined in negative control cultures [30]. All the results were expressed as mean ± Standard Deviation (SD) of the mean and statistically analyzed by the non-parametric Kruskal-Wallis test, using Statistica version 7 (StatSoft South America-Brazil). Differences were considered to be significant at p < 0.05.

### 5. *In vivo* homozygotization assay [24]

**Fig 1:**
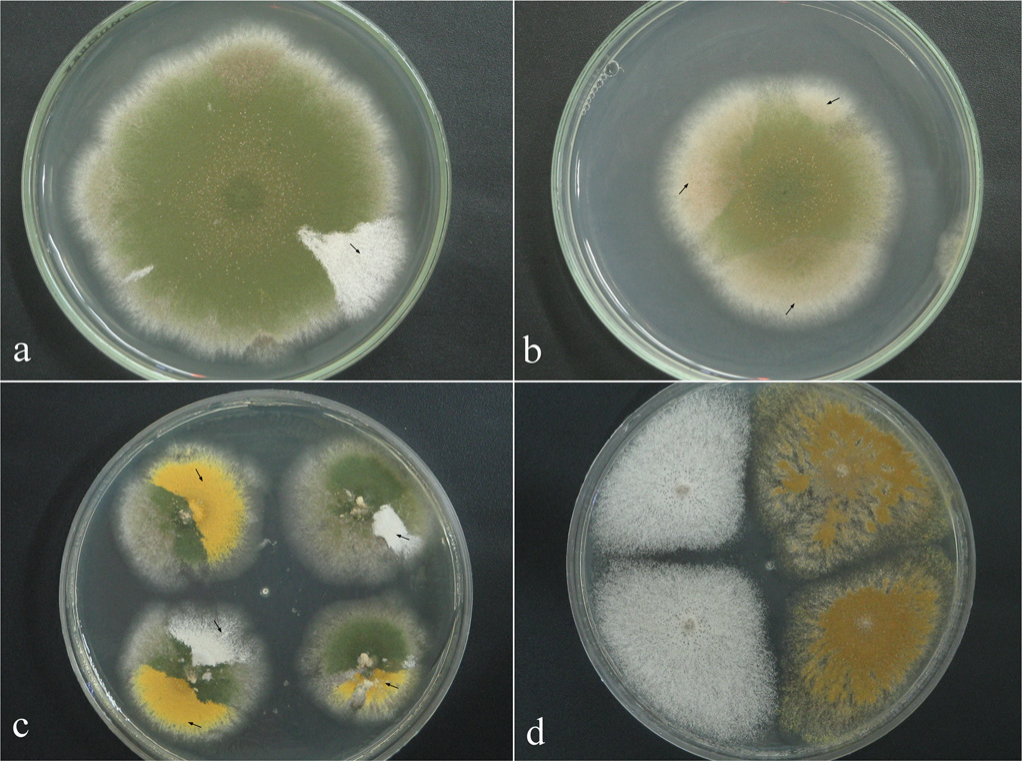
Glibenclamide-treated diploids and their haploid mitotic segregants. **(a)** Mitotic segregant (arrow) derived from the UT448//A757 diploid strain growing in the absence of glibenclamide. **(b)** Origin of the glibenclamide-treated diploids (arrows) in plates containing MM + 10 μM glibenclamide. **(c)** Mitotic segregants (arrows) derived from the 100 μM glibenclamide-treated diploid. **(d)** Haploid (left) and aneuploid (right) segregates derived from the diploid obtained with 10 μM of glibenclamide.

**Fig 2:**
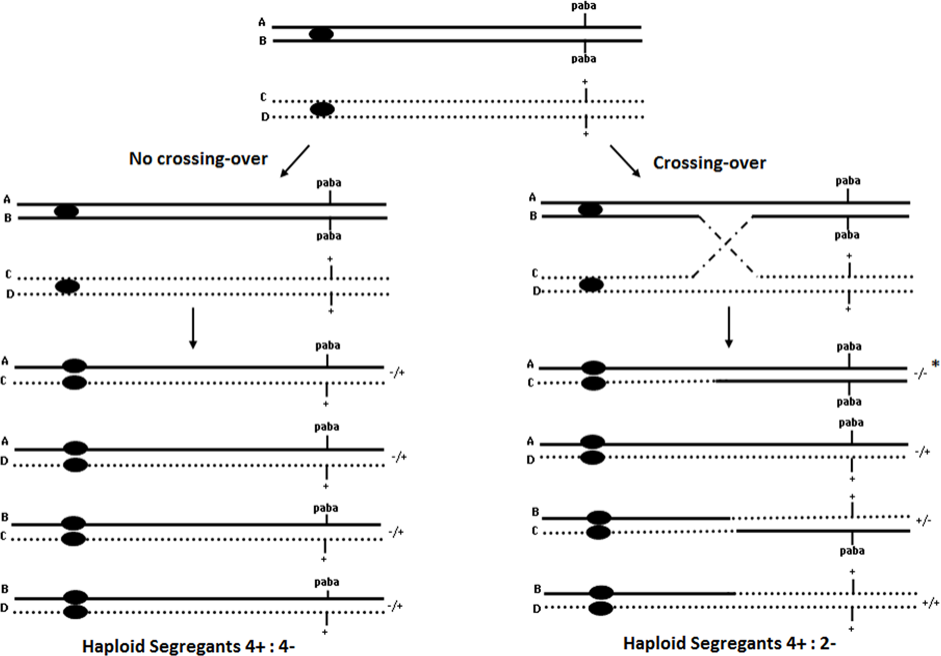
Origin of heterozygous and homozygous diploids caused by mitotic crossing-over between *paba* gene and centromere. (*) Not grown in MM [24].

The master strains (a) A757, with yellow conidia (*yA2*), and nutritional requirements for methionine (*methA17*), and pyridoxine (*pyroA4*), and (b) UT448, with white conidia (*wA2*) and with nutritional requirements for riboflavin (*riboA1*), *p*-aminobenzoic acid (*pabaA124*), and biotin (*biA1*), and resistant to acriflavin (*AcrA1*), were used to form the UT448//A757 diploid strain of *A*. *nidulans* [24, 31]. The UT448//A757 diploid strain, with green conidia is heterozygous for the conidia color markers *yA2* (yellow) and *wA2* (white) and for the nutritional markers and it may grow in Minimal Medium (MM) consisting of Czapek-Dox medium supplemented with 1% (w/v) glucose. When growing on the Complete Medium (CM) [31], the diploid strain may originate auxotrophic mitotic segregants, which are identified as normally growing yellow, green or white sectors on UT448//A757 diploid green colonies. The Supplemented Medium (SM) consisted of MM supplemented with all the nutritional requirements of the strains which form the diploid, except one in each SM type. Solid medium contained 1.5% (w/v) agar. The glibenclamide concentration (100 μM) that induced the production of ROS in human cancer cells [6] and two lower concentrations (1 μM, 10 μM) were used in the cytotoxicity and homozygotization assays. UT448//A757 diploid colonies’ diameters were evaluated for cytotoxicity during six days after incubation at 37°C. The growth rates in the presence (treatment) and in the absence (control, Fig. 1a) of glibenclamide were compared by one-way ANOVA and subsequent Bonferroni’s test, at p < 0.05. All tested glibenclamide concentrations showed no cytotoxicity (results not shown). UT448//A757 diploid strain colonies were grown onto petri plates containing MM (negative control), MM + cytosine arabinoside (0.4 μM, positive control) and MM + glibenclamide (treatment). Plates were incubated for six days at 37°C and then visually inspected for diploid sectors arising on the diploid strains’ colonies. All the treatments with glibenclamide produced morphologically identifiable diploid sectors for each test concentration (Fig. 1b). Diploids were homozygous (+/+) or heterozygous (+/- or-/+) but not recessive (-/-) for nutritional markers, since the latter cannot grow on MM. The untreated diploid strains (negative control) and those obtained after treatment with glibenclamide (1 μM, 10 μM and 100 μM) and cytosine arabinoside (0.4 μM) were purified on MM, individually transferred to CM plates and processed by spontaneous haploidization. The haploidization process consists of the loss of one member of each chromosome pair through successive mitotic divisions and results in the haploid condition of nuclei. After haploidization, the haploid mitotic segregants (Fig. 1c) were purified in CM and their mitotic stability evaluated in CM + benomyl (0.2 μg/mL). Benomyl, a haploidizing agent, is a strong spindle toxin, leading to disturbance in the mitotic segregation of the chromosomes [32]. Only mitotically stable haploid segregants at the final stage were selected for the recombinogenesis test (Fig. 1d). In the case of phenotypic analyses, the haploid segregants were individually transferred to different SM. Mitotic crossing-over cause homozygotization of heterozygous-conditioned genes. If the glibenclamide induces mitotic crossing-over in the original diploid strain, only heterozygotes (+/- or-/+) or homozygotes (+/+) diploids will develop in MM and the nutritional markers will segregate among the haploids in the proportion of 4+ to 2-. However, if the antidiabetic drug fails to induce crossing-over, the proportion will be 4+ to 4-. This is due to the fact that the initial selection process limits the growth of-/- diploids [24] (Fig. 2). The ratio of prototrophic to auxotrophic segregants is described by the Homozygotization Indice (HI), or rather, an HI equal to or higher than 2.0 indicates recombinogenic effects of the substance test. The recombinogenic potential of the glibenclamide was assessed by comparing the HI rates of the nutritional markers with Yates’s corrected Chi-square test, Contingency Table, using Statistic version 7 (StatSoft South America-Brazil). Differences were considered to be significant at p < 0.05.

## Results

**Fig 3:**
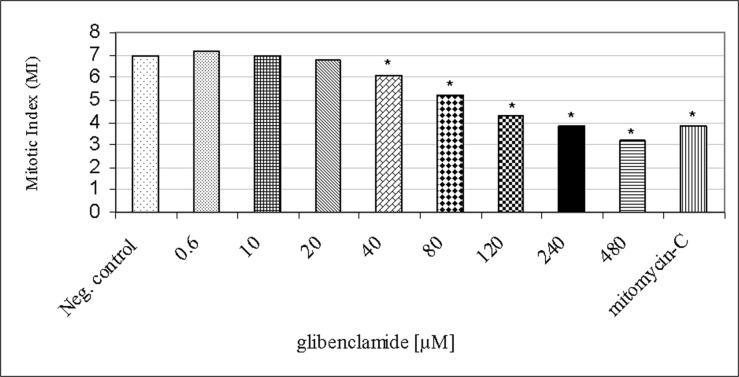
MI rates induced by glibenclamide (0.6 μM, 10 μM, 20 μM, 40 μM, 80 μM, 120 μM, 240 μM and 480 μM) in human lymphocytes. Neg. control = negative control, mitomycin-C (0.3 μM) = positive control. (*) Significantly different from negative control (Kruskal-Wallis test, p < 0.05).

**Fig 4:**
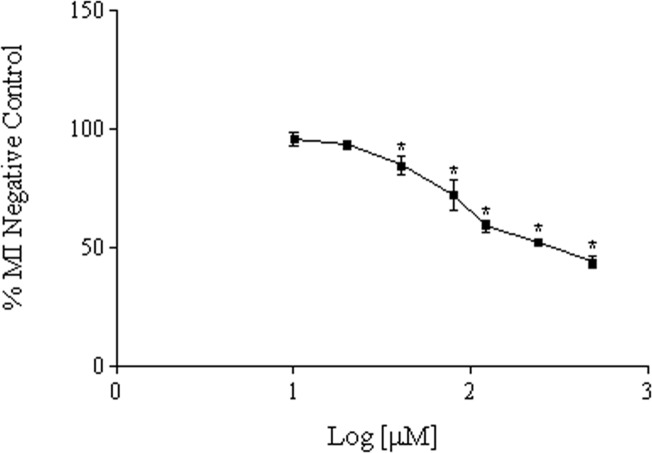
Glibenclamide dose-response curve, showing the percent control MI of the various concentrations tested. The Ordinate shows the MI rates of glibenclamide concentrations (10 μM, 20 μM, 40 μM, 80 μM, 120 μM, 240 μM and 480 μM) expressed as percentage of negative control MI. The abscissa shows the log of glibenclamide concentrations. *Significantly different from negative control (Kruskal-Wallis test, p < 0.05).

Eight concentrations of glibenclamide (0.6 μM, 10 μM, 20 μM, 40 μM, 80 μM, 120 μM, 240 μM and 480 μM) were evaluated by determining of the MI rates. Glibenclamide at 0.6 μM to 20 μM affected neither the normal cell morphology of lymphocytes nor the MI rates. In fact, they were not significantly different from those obtained in the untreated cultures (negative control, p > 0.05; Fig. 3). On the other hand, the MI rates in the other glibenclamide concentrations (40 μM to 480 μM) showed a statistically significant reduction when compared with the negative control (p < 0.05). Glibenclamide at the highest concentration (480 μM) showed a cytotoxicity of approximately 56% when compared to that of the negative control (Figs. 3 and 4).

**Fig 5:**
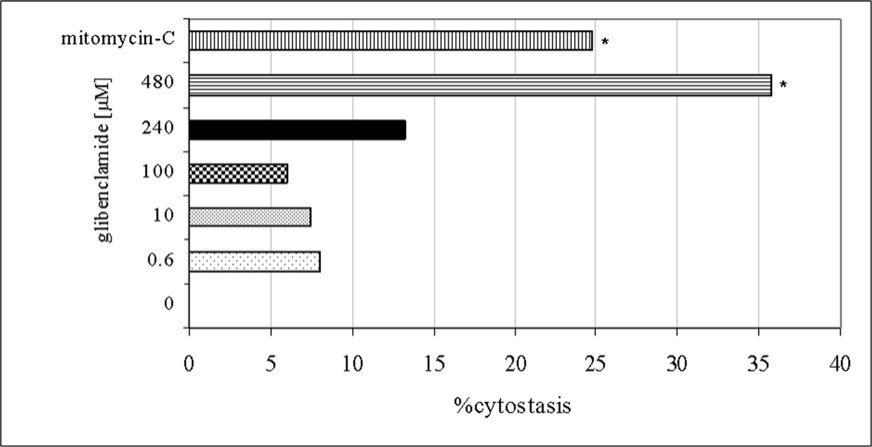
The amount of cytostasis induced by each glibenclamide concentrations (0.6 μM, 10 μM, 100 μM, 240 μM and 480 μM) compared to the negative control. Mitomycin-C (0.3 μM) = positive control. (*) Significantly different from negative control (Kruskal-Wallis test, p < 0.05).

Since the analysis of MI was used as an indicator of glibenclamide cytotoxicity, the glibenclamide concentrations selected for MNvit test were 0.6 μM, 10 μM, 100 μM, 240 μM and 480 μM which produced cytostasis ranging approximately between 6% and 35.75% when compared to that of the negative control (Fig. 5).

**Fig 6:**
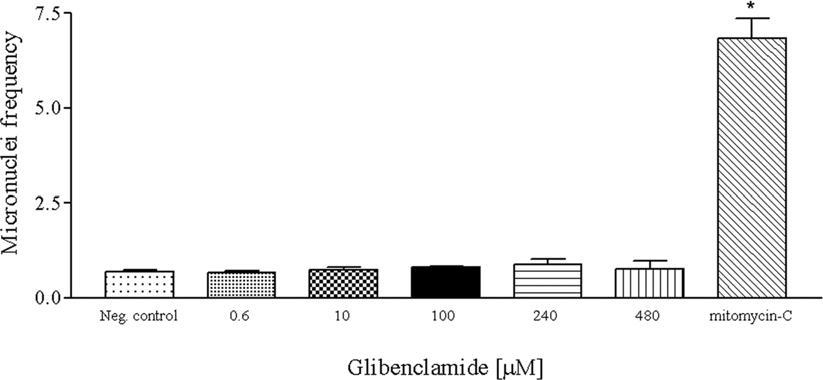
Mean of micronuclei frequencies observed in human lymphocytes cultured *in vitro* after glibenclamide treatment. Neg. control = negative control, mitomycin-C (0.3 μM) = positive control. (*) Significantly different from negative control (Kruskal-Wallis test, p < 0.05).

Whereas micronucleated cells were rare in glibenclamide-treated cells as well as in untreated cells (negative control), mitomycin-C, the positive control, at 0.3 μM concentration, caused a significant rise (p < 0.05) in the number of micronuclei when compared to the negative control. Data on the occurrence of micronucleated cells are shown in Table 1. The frequencies of micronucleated lymphocytes were 0.7% for the negative control and 6.85% for the positive control. When compared to the negative control, glibenclamide at 0.6 μM, 10 μM, 100 μM, 240 μM and 480 μM concentrations failed to exhibit any significant increase in the frequencies of micronucleated cells, which ranged from 0.68% to 0.9% (Fig. 6). Glibenclamide at 480 μM concentration decreased significantly the CBPI values when compared to those of the negative control. Although nuclear buds (NB) and nucleoplasmic bridges (NPB) have been observed in the glibenclamide treated cultures, the results were not statistically significant (Table 1).

**Table 1:** Effect of glibenclamide on micronuclei, nuclear buds and nucleoplasmic brigdes induction in human lymphocytes *in vitro*.

Test Substance	Treatment		BC scored	BCMN (mean ± SD)	Total number of MN	BCNB (mean±SD)	Total number of NB	BCNPB (mean±SD)	Total number of NPB	CBPI (Mean±SD)
	Period (h)	Concentration (μM)								
Negative control	24	-	4000	7.0±0.82	28	4.5±3.87	18	0.75±0.50	3	2.06 ± 0.08
DMSO	24	1%	4000	8.5±1.29	34	6.5±1.29	26	0.25±0.50	1	1.98 ± 0.08
Positive control	24	0.3	4000	68.5±10.02*	274*	12.75±5.1	51	0.5±1.00	2	1.80 ± 0.13
glibenclamide	24	0.6	4000	6.75±0.95	27	6.5±3.00	26	0	0	2.02 ± 0.07
	24	10	4000	7.5±1.29	30	6.0±2.16	24	0	0	1.98 ± 0.06
	24	100	4000	8.0±0.82	32	6.25±3.30	25	0	0	1.98 ± 0.05
	24	240	4000	9.0±2.12	36	7.0±1.82	28	0.5±0.58	2	1.9 ± 0.04
	24	480	4000	7.75±4.35	31	8.0±2.16	32	1.0±1.41	4	1.7 ± 0.18*

BC: binucleated cells; BCMN: binucleated cells with micronuclei; MN: micronuclei; BCNB: binucleated cells with nuclear buds; NB: nuclear buds; BCNPB: binucleated cells with nucleoplasmic bridge; NPB: nucleoplasmic bridges; CBPI: cytokinesis block proliferation index. Positive control: mitomicyn-C.

(*) Significantly different from negative control (non-parametric Kruskall-Wallis test, p < 0.05).

**Fig 7:**
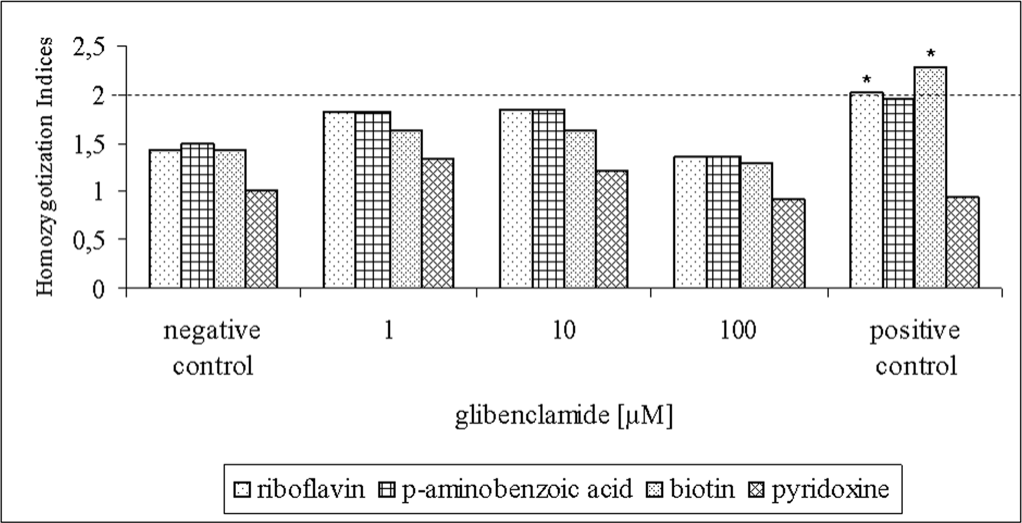
Homozygotization Indices (HI) rates for nutritional markers from UT448//A757 diploid strain after treatment with 1 μM, 10 μM and 100 μM of glibenclamide. Positive control = cytosine arabinoside (0.4 μM). (*) Significantly different from negative control (Contingency Table, Yates Corrected Chi Square, p < 0.05).

The recombinogenic potential of glibenclamide was evaluated by the HI rates for *A*. *nidulans* nutritional markers from chromosomes I (*riboA1*, *pabaA124* and *biA1*) and IV (*pyroA4*). Nine glibenclamide-treated diploids, three diploids treated with cytosine arabinoside and three untreated diploids were selected in MM which did not allow the development of auxotrophic diploids, specifically those which were homozygous for *ribo*, *paba*, *bi*, *meth* and *pyro* markers. Thus, only prototrophic diploids with green conidia were analyzed in the recombinogenic test. As expected, the HI rates for untreated diploids (negative control) were lower than 2.0 for all analyzed markers. On the other hand, cytosine arabinoside (0.4 μM), the positive control, induced HI rates which were higher than 2.0 for the *ribo* and *bi* markers and significantly different (p < 0.05) from the negative control. The HI rates for diploids obtained with all the three concentrations of glibenclamide (1 μM, 10 μM and 100 μM) were lower than 2.0 for all analyzed markers. Data for HI rates for both treated and untreated diploids are shown in Fig. 7.

## Discussion

Current study investigated the mutagenic and recombinogenic potentials of glibenclamide in eukaryotic cells, the latter for the first time. Glibenclamide caused neither cytogenetic toxicity in human lymphocytes cultures nor recombinogenesis in eukaryotic cells when used at therapeutic or higher concentrations. It supported the clinical use of this antidiabetic drug in the treatment of diabetic and cancer patients.

Glibenclamide did not induce significant increase in the frequencies of micronuclei as well as in the number of NB and NPB. Since the MNvit is a screening method to detect structural chromosome damage or changes in the chromosome number [21], data in the current study demonstrated a lack of mutagenic activity of glibenclamide even when used in concentrations several hundred times greater than the plasma concentration. Based on the fact that the cytotoxicity of a genotoxic chemical in a cell culture may trigger cytostasis and cell death, the CBPI rates have been considered an index of cell kinetics and cytotoxicity [30]. Glibenclamide at 480 μM changed the cell-proliferation kinetics, significantly reducing the CBPI value when compared to the negative control. Results agree with those by Ouadid-Ahidouch and Ahidouch [5] which show the anti-proliferative effect of this hypoglycemic drug.

The recombinogenic potential of glibenclamide was evaluated by the homozygotization assay, a bioassay extensively used to detect the genotoxic effects of several chemical agents such as environmental volatile pollutants, herbicides, antidiabetic and cancer chemotherapeutic compounds [31, 33–35]. Mitotic recombination due to non-sister chromatids exchange produces the loss of heterozygozity (LOH) for markers distal to the recombination site [36]. In our analysis, *A*. *nidulans* diploids, homozygous for the nutritional markers (+/+), were not obtained among the glibenclamide-treated diploids. In fact, the heterozygous condition of the nutritional markers (+/- or-/+) was evidenced by the production of prototrophic and auxotrophic segregants during the haploidization of the glibenclamide-treated diploids. The absence of homozygous diploids (+/+) was reflected in the HI rates which were not statistically different from the negative control (non-treated diploids), demonstrating that glibenclamide is devoid of any recombinogenic activity. The antidiabetic drug actually showed no recombinogenic effect even when used in the same concentration (100 μM) that induced DNA fragmentation in recombinant human embryonic kidney cells and production of ROS in gastric cancer cells [9, 37].

Homologous recombination is a process which involves reciprocal exchange of genetic material between homologous chromosomes and promotes genome stability through the precise repair of DNA lesions, including DNA double-strand breaks. There is now abundant evidence that amplification of certain oncogenes and LOH in tumor suppressor genes are important mechanisms involved in cancer initiation and/or progression. Due to mitotic recombination, LOH has been observed in several types of tumor and is a major contributor to the tumorigenic process [17, 38].

A series of *in vitro* and *in vivo* assays have been currently employed to evaluate the genotoxicity of chemical compounds. In addition to the tests utilized in the current study, the *in vivo* somatic mutation and recombination test (SMART), an one-generation test developed to detect LOH due to different genotoxic events (i.e., mitotic recombination, point mutations and chromosomal aberrations) in *Drosophila melanogaster* [39], and the *in vitro* comet assay, a useful approach for assessing DNA damage in eukaryotic cells [40], have been widely used in genotoxicity studies.

The monoterpene 2-methylisoborneol, an odorous substance produced by different groups of heterotrophic microorganisms [41], was recently assessed for genotoxicity using the SMART, the comet and the Cytokinesis Block Micronucleus (CBMN)-cytome assays. Investigations showed that 2-methylisoborneol induced neither mutagenesis nor recombinogenesis in *Drosophila melanogaster* and was not genotoxic in the CBMN-cytome assay using Chinese hamster ovary cells. Positive results were obtained in the comet assay only when 2-methylisoborneol was used at the highest concentration [42].

Recently, our research group evaluated the genotoxic potential of metformin, a hypoglycemic drug also prescribed for the treatment of type 2 diabetes mellitus, using the *in vitro* MNvit and chromosomal aberrations tests in human lymphocytes and the *in vivo* homozygotization assay. Metformin was characterized as genotoxic neither *in vitro* nor *in vivo* [31].

Glibenclamide is an antidiabetic drug with inhibitory effects on the proliferation of different human carcinoma cells, including breast, colon and bladder cancer cells [12, 43, 44]. Many agents used in human cancer chemotherapy, such as cisplatin, have been characterized as inducers of second malignances. It has been recently demonstrated that DNA damages induced by cisplatin may ultimately contribute to the increased incidence of secondary leukemias seen in patients cured of primary malignancies with platinum-based regimens [45]. In the current study, the ability of glibenclamide to cause mitotic crossing-over *in vivo* and mutagenicity *in vitro* was evaluated. Results demonstrated that glibenclamide, in different analysis systems, and in our experimental conditions, was devoid of significant mutagenic and recombinogenic effects. Data suggest that glibenclamide is not a second malignances-inducer. This fact encourages further investigations on the use of this antidiabetic agent as a chemotherapeutic drug and point to the safety usage of glibenclamide for the treatment of type 2 diabetes mellitus, including diabetic patients already taking the drug.
